# Combination Strategies for Immune-Checkpoint Blockade and Response Prediction by Artificial Intelligence

**DOI:** 10.3390/ijms21082856

**Published:** 2020-04-19

**Authors:** Florian Huemer, Michael Leisch, Roland Geisberger, Thomas Melchardt, Gabriel Rinnerthaler, Nadja Zaborsky, Richard Greil

**Affiliations:** 1 Department of Internal Medicine III with Haematology, Medical Oncology, Haemostaseology, Infectiology and Rheumatology, Oncologic Center, Paracelsus Medical University, 5020 Salzburg, Austria; f.huemer@salk.at (F.H.); m.leisch@salk.at (M.L.); t.melchardt@salk.at (T.M.); g.rinnerthaler@salk.at (G.R.); 2 Salzburg Cancer Research Institute-Laboratory for Immunological and Molecular Cancer Research (SCRI-LIMCR), 5020 Salzburg, Austria; r.geisberger@salk.at (R.G.); n.zaborsky@salk.at (N.Z.); 3 Cancer Cluster Salzburg, 5020 Salzburg, Austria

**Keywords:** kinase inhibitor, vaccination, CAR-T cell, radiomics, PD-1, PD-L1, tumor neoantigen, HLA, resistance mechanism, T cell exhaustion

## Abstract

The therapeutic concept of unleashing a pre-existing immune response against the tumor by the application of immune-checkpoint inhibitors (ICI) has resulted in long-term survival in advanced cancer patient subgroups. However, the majority of patients do not benefit from single-agent ICI and therefore new combination strategies are eagerly necessitated. In addition to conventional chemotherapy, kinase inhibitors as well as tumor-specific vaccinations are extensively investigated in combination with ICI to augment therapy responses. An unprecedented clinical outcome with chimeric antigen receptor (CAR-)T cell therapy has led to the approval for relapsed/refractory diffuse large B cell lymphoma and B cell acute lymphoblastic leukemia whereas response rates in solid tumors are unsatisfactory. Immune-checkpoints negatively impact CAR-T cell therapy in hematologic and solid malignancies and as a consequence provide a therapeutic target to overcome resistance. Established biomarkers such as programmed death ligand 1 (PD-L1) and tumor mutational burden (TMB) help to select patients who will benefit most from ICI, however, biomarker negativity does not exclude responses. Investigating alterations in the antigen presenting pathway as well as radiomics have the potential to determine tumor immunogenicity and response to ICI. Within this review we summarize the literature about specific combination partners for ICI and the applicability of artificial intelligence to predict ICI therapy responses.

## 1. Introduction

It has been recognized for a long time that tumor cell evasion from the immune system is a hallmark of malignant cancers [1] and several mechanisms have been identified by which tumor cells shape an immunosuppressive microenvironment, that are reviewed elsewhere [2,3,4]. One of the best-studied and most relevant mechanisms is the suppression of T cells through activation of negative regulatory pathways by tumor cells, so-called immune-checkpoints. Several immune-checkpoint molecules have been identified in the last years with cytotoxic T-lymphocyte protein 4 (CTLA-4) and programmed cell death protein 1 (PD-1) being the best studied systems [5]. These molecules play a crucial role in controlling the physiological immune response and in preventing over-activation of the immune system [6,7].

**Figure 1:**
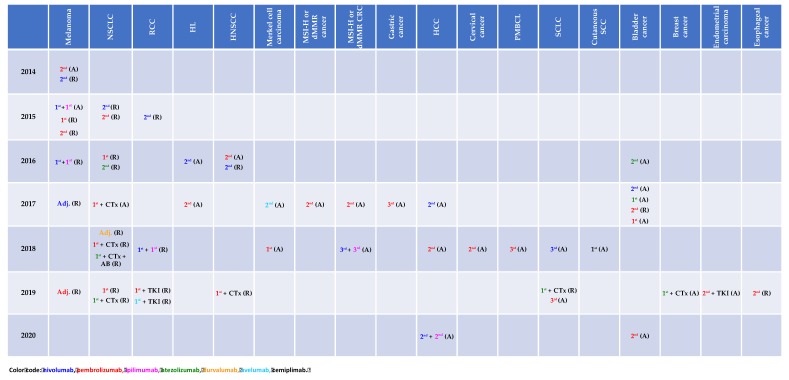
Immune-checkpoint inhibitor approval status by the Food and Drug Administration (access date: 03/13/2020). A: accelerated, AB: antibody, CTx: chemotherapy, dMMR: mismatch repair deficiency, HCC: hepatocellular carcinoma, HL: Hodgkin’s lymphoma, HNSCC: head and neck squamous cell carcinoma, MSI-H: microsatellite instability, NSCLC: non-small cell lung cancer, PMBCL: primary mediastinal B cell lymphoma, R: regular, RCC: renal cell carcinoma, TKI: tyrosine kinase inhibitor.

A magnitude of clinical trials investigating immune-checkpoint inhibitors (ICI) as monotherapy, combination therapy or in combination with cytotoxic agents as well as with targeted therapy has demonstrated improved clinical outcome across various types of cancer and in turn led to the respective approval status by the Food and Drug Administration (FDA) as depicted in Figure 1.

Although immune-checkpoint blockade derives long-term overall survival (OS) in a subset of cancer patients [8,9], identification of patients who will not benefit from ICI remains challenging. Extensively investigated biomarkers such as programmed cell death-ligand 1 (PD-L1) [10,11,12,13] help predicting clinical outcome with ICI whereas negativity does not exclude responses [14,15]. Tumor immunogenicity is a prerequisite for reversing T cell exhaustion by ICI. Although alterations in the antigen presenting machinery have a relevant impact on the therapeutic success of immune-checkpoint blockade [16,17], the latter findings have not influenced clinical decision-making so far. Radiomics—extracting information about tumor biological processes from imaging studies—may serve as an alternative to tumor tissue-based biomarkers and may facilitate response prediction to ICI. Apart from combining ICI with chemotherapy, other ICI or monoclonal antibodies, growing evidence provides the rationale for combination strategies with kinase inhibitors [15,18] or tumor-specific vaccinations [19]. While treatment with chimeric antigen receptor (CAR)-T cells, T cells genetically modified ex vivo to express a new surface antigen receptor, led to unprecedented response rates in hematologic malignancies such as relapsed/refractory B cell acute lymphoblastic leukemia (B-ALL) [20] and diffuse large B cell lymphoma (DLBCL) [21,22], therapeutic success in solid cancers is poor [23,24]. Up-regulation of immune-checkpoints and in turn ineffective T cell function have been identified as primary and secondary resistance mechanisms to CAR-T cell therapy and therefore provide the rationale for combinations with ICI [25,26].

Within this review, we summarize and discuss the biological background of immune-checkpoints (with focus on PD-1), specific promising combination partners for immune-checkpoint blockade (kinase inhibitors, tumor-specific vaccinations, CAR-T cells) and response prediction to ICI by artificial intelligence (with focus on the antigen presenting pathway) including the applicability of radiomics.

## 2. Programmed Cell Death Protein 1 (PD-1) and its Key Role in T Cell Exhaustion

In search for proteins that mediate programmed cell death in T cells upon cytokine deprivation, around 30 years ago Ishida and colleagues identified a protein they termed PD-1, which was inducibly expressed in T cell lines undergoing apoptosis [27]. Structurally, the 50–55-kDa type I transmembrane glycoprotein PD-1 is a monomeric member of the immunoglobulin gene superfamily with an IgV domain homologous to CD28, CTLA-4 and ICOS in the extracellular region. While further research did not confirm a direct participation of PD-1 in programmed cell death of T cells, knockout mice revealed that loss of PD-1 increases the risk of severe T cell mediated autoimmune pathologies and lupus-like syndromes, showing that PD-1 negatively regulates T cell immune responses as an “immune-checkpoint” molecule [28,29,30]. It soon turned out that PD-1 on T cells is important for induction of peripheral but not central T cell tolerance. While interaction of T cells with resting dendritic cells results in tolerance induction, this interaction leads to efficient T cell priming in the absence of PD-1 [31]. In line with this, PD-1 was found to be a key molecule in chronic viral infections in mice. Mice infected with different lymphocytic choriomeningitis virus strains can either suffer an acute infection which is efficiently cleared within weeks or otherwise, the virus is not effectively combated and persists chronically [32]. In the latter case, high numbers of virus specific PD-1 positive T cells accumulate in the host and blocking PD-1 (by blocking antibodies or PD-1 knockout) results in T cell reactivation and clearance of the virus [33]. Based on these experiments, the term T cell exhaustion was coined for antigen specific, primed, PD-1 positive T cells, unable to fight off target cells. In search for ligands for PD-1, two transmembrane glycoproteins were discovered, termed PD-L1 and PD-L2 [34,35]. Unlike PD-1, which is expressed only in distinct immune cell subsets [36], PD-L1/2 are expressed on a wide variety of tissues (Table 1). Particularly, high PD-L1 expression was noticed on some tumor cells and it was shown that its expression suppresses the cytolytic activity of cancer-specific T cells. Analogous to virus experiments, blocking PD-1/PD-L1 interactions can result in the reinvigoration of efficient anti-cancer immune responses, both in mouse models and in patients, proving that T cell exhaustion significantly contributes to immunological tolerance towards tumor cells [37,38,39]. While PD-L1/2 have only short cytoplasmic tails with signaling competence so far only reported in B cells [40], the cytoplasmic domain of PD-1 comprises two conserved signaling motifs, immunoreceptor tyrosine-based inhibitory motif (ITIM) and immunoreceptor tyrosine-based switch motif (ITSM). Generally, PD-L1/2 interaction with PD-1 induces phosphorylation of ITIM and ITSM, leading to recruitment of the phosphatases SHP-1 and SHP-2. Subsequent dephosphorylation of the T cell receptor (TCR) activation signals CD-3ζ and zeta chain-associated protein kinase 70 (ZAP70) leads to inhibition of the downstream phosphatidylinositol 3-kinase (PI3K)/AkT/Ras signaling pathway and shuts down cytokine production and effector activities [41,42,43]. Importantly, PD-1 expression is not restricted to exhausted T cells but generally up-regulated upon T cell activation and expressed in various T cell subsets, such as regulatory T cells (Treg), T follicular helper (TFH) cells, T follicular regulatory (TFR) cells and memory T cells. In addition, it is expressed in several other cell types including B cells, natural killer (NK) cells, some myeloid cells and cancer cells upon activation [36]. Consequently, downstream signaling pathways may be different in the respective cell subsets and vastly depend on co-signals from the microenvironment as well as differentiation, metabolic and hypoxic states. Notably, profiling of exhausted T cells revealed expression of several other exhaustion-related immune-checkpoint receptors, such as T cell membrane protein 3 (TIM-3) or lymphocyte-activation gene 3 (LAG-3) [44] and the outcome of ICI therapies may depend on the exact composition of PD-1 positive immune cell subsets as well as their complex spatiotemporal dynamics in their interaction with tumor cells. Hence, to maximize durable clinical responses to ICI, it will be crucial to find effective combination treatments, which are discussed in this review.

## 3. Kinase Inhibitors as Combination Treatments to Increase T Cell Activation

**Figure 2:**
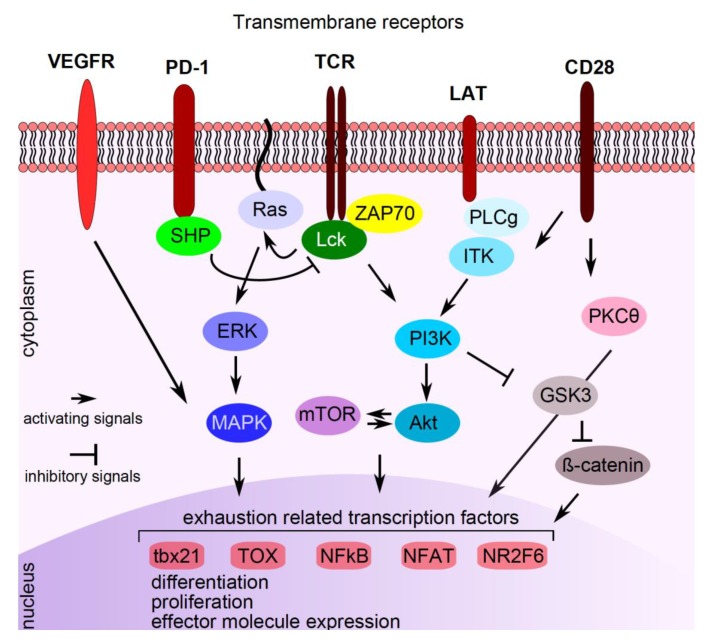
Pathways interfering with PD-1 signaling. Signaling compounds are indicated in the cytoplasm, transcription factors/repressors are indicated in the nucleus. See text for explanations. VEGFR: vascular endothelial growth factor receptor, PD-1: programmed cell death protein 1, TCR: T cell receptor, LAT: linker for activation of T cells, CD: cluster of differentiation, SHP: small heterodimer partner, Lck: lymphocyte-specific protein tyrosine kinase, ZAP70: zeta chain-associated protein kinase 70, PLCg: phospholipase C gamma 1, ITK: interleukin-2 inducible T cell kinase, ERK: extracellular signal-regulated kinase, PI3K: phosphatidylinositol 3-kinase, PKCӨ: protein kinase C theta, MAPK: mitogen-activated protein kinase, mTOR: mechanistic target of rapamycin, Akt: protein kinase B, GSK3: serine/threonine kinase glycogen synthase kinase 3, tbx21: T-box transcription factor 21, TOX: thymocyte selection-associated high mobility group box protein TOX, NFKB: nuclear factor kappa-light-chain-enhancer of activated B cells, NFAT: nuclear factor of activated T cells, NR2F6: nuclear receptor subfamily 2 group F member 6.

Although immune-checkpoint inhibition by blocking antibodies efficiently unleashes effective anti-cancer immune responses in some patients, current studies show that targeting T cells by additional (anti-cancer) compounds may potentiate immune-checkpoint therapies. In this context, drugs were shown either to modulate expression of inhibitory receptors on T cells or to interfere with T cell function or differentiation, both synergizing with immune-checkpoint therapies (Figure 2). The serine/threonine kinase glycogen synthase kinase 3 (GSK-3) is a central regulator of PD-1 transcription in CD8+ T cells. Silencing or pharmacological inhibition of GSK-3 in mice resulted in up-regulation of T-box transcription factor 21 (tbx21) in CD8+ T cells, which in turn led to down-regulation of PD-1 and to enhanced cytolytic CD8+ T cell function [46]. In murine cancer models, GSK-3 inhibition was similarly effective as PD-1/PD-L1 blockage in reinvigorating anti-cancer immunity and a mild synergistic effect was noticed [47,48].

The mechanistic target of rapamycin (mTOR) kinase functions in the context of multiprotein signaling complexes mTORC1/2, which are implicated in diverse metabolic, stress and immunological pathways [49]. Although mTOR inhibition by sirolimus or everolimus is largely immune suppressive and used to impede host versus graft rejection after organ transplantation, intermediate doses of the mTOR inhibitor vistusertib selectively promote effector T cell function and potentiate anti-PD-1, anti-PD-L1 and anti-CTLA-4 therapy in a colorectal mouse tumor model [50].

Interference with cell cycle progression has become an attractive concept for cancer therapy and recently, several cell cycle inhibitors, particularly inhibitors of cyclin dependent kinase 4 and 6 (CDK4/6) (abemaciclib, palbociclib and ribociclib) have been approved for advanced estrogen receptor positive breast cancer [51,52,53]. However, CDK4/6 inhibitors have additional immune activating effects, by increasing antigen presentation in cancer cells and by mediating increased nuclear factor of activated T cells (NFAT) activation in response to TCR engagement and repressed DNA methyltransferase 1 in T cells, which augments anti-cancer immunity upon PD-1 blockade [54,55,56]. Ibrutinib, a Bruton’s tyrosine kinase (BTK) inhibitor, has become standard of care for relapsed and high-risk chronic lymphocytic leukemia (CLL) patients [57]. Although ibrutinib was developed to specifically inhibit BTK dependent B cell receptor (BCR) signaling, it also binds interleukin (IL)-2-inducible T cell kinase (ITKs) in T cells, which leads to increased T cell numbers and function due to impaired activation-induced cell death through ITK inhibition [58] and due to Th1 polarization [59]. Concomitantly, in mouse experiments, ibrutinib enhanced T cell anti-tumor immunity [60], prompting the initiation of a phase 2 study on ibrutinib and PD-1 blockade (pembrolizumab) combination therapy in high-risk CLL (ClinicalTrials.gov Identifier: NCT03514017). In line with this, another BCR signaling inhibitor, acting on PI3K (idelalisib) was also shown to decrease proliferation and effector function of Tregs in vitro. This effect was mediated by inhibition of the PI3K/Akt/nuclear factor kappa-light-chain-enhancer of activated B cells (NFKB) axis, whereupon a synergistic effect of idelalisib with immune-checkpoint inhibition was proposed [61,62].

Recently, the transcription factor TOX was identified to be responsible for driving an exhaustion specific transcriptional profile in T cells. TOX is activated by vascular endothelial growth factor (VEGF)-A upon binding to VEGF receptor (VEGFR) on T cells and inhibition of VEGFR potentiated anti-tumor immunity in mice treated with ICI [63]. Hence, specific kinase inhibitors interfering with VEGF-A/VEGFR downstream signaling could act synergistically with PD-1 blockade in reinvigorating exhausted T cells. In line with this, a specific mitogen-activated protein kinase (MAPK) inhibitor (G-38963, which is similar to cobimetinib) counteracts TCR-induced apoptosis of tumor infiltrating cytotoxic T cells in mice, thereby potentiating anti-tumor immunity. Moreover, combination with anti-PD-L1 treatment results in durable and synergistic tumor regression [64].

Finally, the transcriptional repressor nuclear receptor subfamily 2 group F member 6 (NR2F6) was recently found to be an additional key player of fine-tuning of T cell effector functions. NR2F6 directly occupies promoter regions of important cytokine gene loci, thereby impeding activation-induced binding of NFAT/AP-1 transcription factors [65]. In mouse studies, it could be shown that loss of NR2F6 leads to enhanced T cell activation upon PD-1 blockade and to increased tumor eradication [66]. As NR2F6 is inactivated by protein kinase C (PKC)-theta dependent phosphorylation, specific compounds promoting PKC-theta activity or interfering with NR2F6 dephosphorylation may be useful to potentiate immune-checkpoint therapies [65].

## 4. Combination of Vaccination Strategies with Immune-Checkpoint Blockade

Despite the success of vaccinations against microbes or viral diseases therapeutic vaccinations against cancer cells have not yielded similar success so far. The major obstacle is to achieve a strong enough immune response, which kills millions of tumor cells to achieve a clinical benefit in an exhausted immune system, which is common in cancer patients. Therefore, vaccinations against hepatitis B virus and human papillomavirus as common causes of cancer are the only effective cancer vaccinations so far.

Nevertheless, several improvements on the way to an effective therapeutic cancer vaccination have been achieved in the last decade. Tumor-associated antigens, which are self-proteins that are abnormally expressed by malignant cells, have been used as target antigens in the past. Neoantigens arising from mutations or oncogenic viral antigens may represent more specific and more efficient targets for vaccination strategies [67]. Due to the immune-stimulating effect, ICI may appear as the ideal combination partner for a vaccination against malignant cells to overcome the exhaustion of the patients’ immune system and possible evasion strategies of the cancer cells. Several preclinical studies could demonstrate this synergistic effect of ICI and vaccination so far [68,69,70] and recently a clinical phase II trial showed the feasibility and efficacy in patients with incurable human papillomavirus 16–related cancer [19]. The combination of nivolumab and the vaccine ISA101 targeting the viral proteins E6 and E7 resulted in encouraging progression-free survival (PFS) and OS in 24 patients compared to already published efficacy data of ICI in similar populations. Nevertheless, as in all other ICI trials before, the majority of patients had no response to immunotherapy in this trial resulting in a median PFS of 2.3 months. Therefore, there is great interest in co-stimulatory molecules expressed on T cells of the patients e.g.,: CD28, ICOS, CD27, 4-1BB, OX40 and CD40L, which may enhance immune response [71]. This has already been shown in preclinical models for agonist OX40 or anti-CD40 antibodies, where the addition to the combination of an anti-CTLA-4 antibody and a vaccine enhanced tumor response in a mouse model [72,73].

## 5. Augmenting Immune Response by Combining CAR-T Cell Therapy and Immune-Checkpoint Blockade

CAR-T cells are a form of cellular immunotherapy, where T cells are genetically modified ex vivo to express a new surface antigen receptor [74]. Most currently used CAR constructs consist of a single-chain variable fragment (scFV) antigen-recognition domain of an antibody linked to a CD3-derived T cell activation domain and a costimulatory domain (most commonly CD28, 4.1BB, or both) [74]. This allows for major histocompatibility complex (MHC) independent tumor cell recognition and killing.

CAR-T cells targeting the CD19 surface antigen expressed in various B cell malignancies have led to unprecedented results in B-ALL and DLBCL, which resulted in the approval of Axicabtagene ciloleucel (Yescarta^®^) and Tisagenlecleucel (Kymriah^®^) for relapsed/refractory B cell lymphomas and B-ALL [20,21,22]. The overall response rate (ORR, i.e., patients achieving a complete (CR)- or partial (PR) remission) in clinical trials with anti-CD19 CAR-T cells for aggressive B cell lymphomas and B-ALL ranges from 52% to 83% [20,21,22]. However, the ORR in patients with CLL treated with anti-CD19 CAR-T cells is substantially lower at about 30% [75], indicating that targeting the same antigen in different malignancies results in heterogeneous therapy responses probably due to the unique nature and microenvironment of different tumor types. CAR-T cells have also been tested in various solid tumors (i.e., against human epidermal growth factor receptor 2 (HER2) or mesothelin) [23,24], however, the results have been far less promising compared to hematologic malignancies, indicating that solid tumors can escape and/or suppress CAR-T cells. There are a bundle of steps for successful CAR-T cell treatment: (1) CAR-T cells have to migrate (i.e., home) to the tumor site (2) recognize a tumor specific antigen (3) exert an immune response against the tumor cell to facilitate killing (4) resist immunosuppressive signals in the tumor microenvironment and (5) persist for a certain period of time for long term disease control. There is an accumulating body of evidence, that the immunosuppressive tumor microenvironment suppresses tumor infiltrating lymphocytes (TILs) and also CAR-T cells. Here we want to summarize mechanisms within the tumor microenvironment that hinder CAR-T cell therapy and strategies to overcome these.

### 5.1. Mechanisms of Resistance to CAR-T Cell Therapy within the Tumor Microenvironment

Several different tumor types express PD-L1 either due to up-regulation after mutations in the PD-L1 gene (*CD274)* or as a result of adaptive up-regulation after stimulation with inflammatory cytokines (i.e., interferon-gamma (IFNγ)) present in the microenvironment [76,77]. Binding of PD-L1 to PD-1 generates an inhibitory signal that attenuates the activity of T cells leading to an exhausted phenotype [78,79]. Exhausted T cells are characterized by loss of effector and memory phenotypes, inability to produce cytokines like IFNγ, tumor necrosis factor alpha (TNFα) and IL-2 that inhibits effector functions [78,80].

CAR-T cells, like their physiologic counterparts, express these checkpoint molecules and are therefore equally prone to immunosuppressive signals. Early evidence of this hypothesis was published by Beatty et al. in 2014 [26]. In a mesothelioma mouse model treatment with anti-mesothelin CAR-T cells did not lead to objective responses. After ruling out antigen loss on the tumor cells or lack of CAR-T cell infiltration into the tumor they observed that the CAR-T cells harvested from the tumor site had lost their cytotoxic potential in vitro (i.e., lack of IFNγ production). This was reversible by resting the CAR-T cells ex vivo for 24 h away from the tumor. The CAR-T cells displayed increased expression of the checkpoint molecules PD-1, TIM-3 and LAG-3, which was also reversible after resting the cells ex vivo. These results indicate that CAR-T cells become exhausted and hypofunctional after prolonged exposure to tumor cells due to suppression via checkpoint pathways. Moon et al. confirmed these observations in similar experiments. They injected mesothelioma tumor cell lines into the flanks of NSG mice and treated the mice with anti-mesothelin second generation CAR-T cells. They observed regression of tumor growth but no cures. After excluding antigen loss or lack of CAR expression, they could show that CAR-T cells after antigen encounter in vivo where no longer able to kill mesothelin positive tumor cells in vitro. CAR-T cells that had been exposed to the antigen in vivo, showed a significant up-regulation of PD-1, LAG-3 and TIM-3 indicating CAR-T cell exhaustion [25]. Cherkassky et al. injected anti-mesothelin CAR-T cells into the pleura of mesothelin positive tumor bearing mice and then performed ex vivo stimulation of harvested tumor infiltrating CAR-T cells. Pre-infusion CAR-T cells were used as control. Compared to the control, CAR-T cells exposed to the antigen in vivo had lower levels of cytolytic function and displayed decreased Th1 cytokine secretion in vitro. They could also show that tumor infiltrating CAR-T cells in mice with progressive tumors had high levels of PD-1, TIM-3 and LAG-3 expression indicating that an immunosuppressive microenvironment leads to CAR-T cell hypofunction and favors tumor escape [81]. Taken together, these studies indicate, that CAR-T cells display an exhausted phenotype after prolonged antigen binding in vivo. Gargett et al. evaluated, whether CAR-T cells might already show an exhausted phenotype before infusion. Therefore, they tracked the expression of CD25, CD69, PD-1 and LAG-3 during the manufacturing process of disialoganglioside (GD2) specific CAR-T cells. They observed an up-regulation of PD-1 and LAG-3 upon viral transduction, which declined to normal levels when the cells were cryopreserved. After thawing and in vitro re-stimulation with either anti-CD3/CD28 antibodies or CAR specific antibodies, they observed that re-stimulation via the CAR receptor resulted in higher up-regulation of PD-1 than via CD3/CD28, however, this did not result in a decrease in cytokine production. This shows that GD2 specific CAR-T cells are not functionally exhausted before infusion. When co-culturing the GD2 specific CAR-T cells with melanoma cell lines repetitively, the authors found that the percentage of viable CAR-T cells decreased with each stimulation. Co-cultering with pembrolizumab saved the CAR-T cells from activation-induced cell death, indicating a protective effect of ICI on CAR-T cell viability. Excitingly, when stimulating CAR-T cells in vitro, the authors also found PD-L1 expression on days 3 to 7 after stimulation. The PD-L1 positive cells had lower PD-1 and LAG-3 expression than PD-L1 negative CAR-T cells. When they analyzed blood samples from patients with melanoma treated with the anti-GD2 CAR-T cells in the CARPETS phase I study, they found that, compared to the infused CAR-T cell product, harvested CAR-T cells had up-regulated PD-1 and PD-L1, while normal peripheral CD8+ T cells from the same patient had normal PD-1 expression. They concluded that antigen encounter via the CAR receptor leads to an exhausted phenotype with an associated lack of effector function [82].

Zolov et al. compared the effects of PD-1 signaling of different CAR-T cells. They produced three different CD123 targeting T cells (one with a 4.1BB costimulatory domain, one with CD28 and one without costimulatory domain). They co-cultured these cells with CD123 and PD-L1 positive acute myeloid leukemia (AML) cell lines and found that the CAR-T cells with the CD28 costimulatory domain showed diminished proliferative capacity and cytokine production as compared to the other CAR constructs, indicating that CD28 CAR-T cells might be more prone to exhaustion than their 4.1BB counterpart [83].

All these preclinical models were further corroborated by translational research in the major clinical trials with CD19 CAR-T cells. As an example, Schuster et al. analyzed outcomes of patients with relapsed/refractory aggressive lymphoma from the JULIET trial according to pre-therapeutic CAR-T cell biomarkers. They were able to show, that patients with the highest PD-1/PD-L1 interaction scores as well as patients with the highest percentage of LAG-3 positive T cells had no or short responses and no long-term cures were observed in this cohort of patients [1].

Fraietta et al. reported findings of 41 CLL patients treated with CD19 CAR-T cells. They were able to show that CR patients, compared with PR or non-responding patients, had significantly lower percentages of CAR-T cells with a CD8+PD-1+ phenotype. CD19 CAR-T cells with co-expression of PD-1 and LAG-3 or TIM-3 were associated with poor responses, whereas individuals who had complete and durable remissions were infused with products containing significantly lower frequencies of these cells [84].

To sum up, there is a robust body of evidence from preclinical models and translational research, that CAR-T cell function and persistence can be suppressed by the engagement of checkpoint molecules. In order to cure more patients with this exciting new treatment, strategies to overcome CAR-T cell hypofunction have been explored.

### 5.2. Overcoming Resistance

The discovery of immune-checkpoints and the subsequent development of checkpoint inhibitors against PD-1, PD-L1 or CTLA-4 has revolutionized immune-oncologic treatment approaches in the last years. As mentioned earlier, suppression of CAR-T cells by the tumor microenvironment leading to an exhausted or senescent phenotype seems to play a major role in treatment failure. Therefore, several approaches to augment the immune response to CAR-T cells via different ways of checkpoint inhibition have been explored.

### 5.3. CAR-T Cells Combined with Infused Checkpoint Inhibitors

Back in 2013 John et al. already hypothesized that the combination of PD-1 blockade with CAR-T cells could overcome immunosuppression by the microenvironment. Using anti-HER2 CAR-T cells in combination with a PD-1 blocking antibody in a mouse xenograft breast cancer model, they were able to show that the CAR-T cells up-regulate PD-1 after binding to the tumor cells. Mice treated with the combination of CAR-T cells and the PD-1 antibody displayed the strongest reduction in tumor mass and had the longest survival compared to either treatment alone. On a molecular level the combination of the PD-1 antibody and the CAR-T cells led to increased IFNγ and granzyme B production, indicating enhanced effector cell function [85]. Three years later, Cherkassky et al. reported similar findings in an orthotopic mouse model of pleural mesothelioma. They injected subsequently lower doses of mesothelin-specific CAR-T cells (with either a 4.1BB or CD28 costimulatory domain) into the pleura and observed increasing CAR-T cell exhaustion and decreasing cytolytic function at lower CAR-T cell doses. The repeated antigen encounter led to a decrease in effector mechanisms due to up-regulation of the immune-checkpoints PD-1, TIM-3 and LAG-3. In order to overcome this exhaustion, they injected a PD-1 antibody into the peritoneum on day 30 after CAR-T cell treatment, which led to relevant tumor shrinkage. To circumvent repeated antibody infusions, they genetically engineered the CAR-T cell with either small hairpin (sh)RNA blockade or PD-1 CAR-T cells dominant negative receptors to create PD-1 resistant CAR-T cells. Treatment with the PD-1 resistant CAR-T cell led to enhanced tumor burden control and increased survival compared to non-engineered CAR-T cells [81].

Yin et al. tested humanized IL-13Ra2 targeting second generation CAR-T cells in combination with different checkpoint blockades (anti-PD-1, anti-CTLA-4 and anti-TIM-3) in comparison with humanized epidermal growth factor receptor variant III (EGFRvIII) CAR-T cells in a glioma animal model. In an orthotopic mouse model, they injected glioma cells that expressed both IL-13Ra2 and EGFRvIII. When they looked at the expression levels of PD-1, CTLA-4 and TIM-3 on the different CAR-T cells, they observed a different up-regulation after target binding (i.e., CTLA-4 expression was higher in IL-13Ra2 targeting CAR-T cells than in the EGFRvIII CAR-T cells), indicating, that CAR-T cells targeting different antigens rely on different checkpoint molecules. In line with this observation, they were able to show that the combination of the IL-13Ra2 CAR-T cells with a CTLA-4 checkpoint inhibitor resulted in significantly better tumor killing as compared with the combination of a CTLA-4 antibody with the EGFRvIII CAR-T cell [86].

These mechanistic insights have generated a broad range of clinical studies evaluating CAR-T cells in combination with infused ICI.

In 2017, Heczey et al. reported on a phase I clinical trial with anti-GD2 CAR-T cell therapy in patients with neuroblastoma. Eleven patients with a median age of 6.5 years with relapsed or refractory neuroblastoma were treated in three cohorts. Cohort one only received the GD2 CAR-T cells, cohort two received a prior lymphodepletion with cyclophosphamide/fludarabine and cohort three received the same lymphodepletion and pembrolizumab on days 1 and 21. They were able to show increased T cell expansion in the cyclophosphamide/fludarabine cohort, but no effect of the ICI on CAR-T cell expansion was seen. In addition, patients in cohorts two and three had better OS compared to cohort one. Differences between groups 2 and 3 were not reported, probably due to the small sample size [87].

In the same year, anecdotal evidence regarding the efficacy of this combination approach was published by Chong et al. They reported on a 35-year old male with relapsed DLBCL treated with a CD19 CAR (4.1BB costimulatory domain) who showed progression within one month after CAR-T cell infusion. As a result of high PD-L1 expression within the tumor, pembrolizumab was given on day 26 and the patient achieved a remission with pembrolizumab continued every three weeks for one year. The infusion of pembrolizumab led to an increase in CAR-T cell numbers and a decreased expression of PD-1 on CAR-T cells [88].

In the same year Maude et al. reported on four children with relapsed B-ALL that did not show a sufficient response to anti-CD-19 CAR-T cell treatment, who were treated with pembrolizumab. Pembrolizumab treatment resulted in a prolonged detection of circulating CAR-T cells and led to non-lasting objective responses [89].

At the 2018 ASH meeting, Li et al. reported on their single institution experience with the combination of checkpoint blocking antibodies and anti-CD19 CAR-T cells at the Children’s Hospital of Philadelphia. Fourteen patients with relapsed B-ALL or B-lymphoblastic lymphoma, who demonstrated early CAR-T cell loss or lack of response received an ICI no sooner than 14 days after CAR-T cell infusion. Responses were observed in patients with early B cell recovery and extramedullary disease, with some patients displaying ongoing tumor control with ongoing pembrolizumab infusions [90].

At the 2019 ASH meeting Ardeshna et al. reported on the first results of the Alexander trial. Here, patients with relapsed/refractory DLBCL were treated with a bicistronic anti-CD19/anti-CD22 CAR-T cell (AUTO-3) followed by pembrolizumab for three doses every three weeks starting at day 14 after CAR-T cell infusion. Of the 24 patients that have been enrolled, 11 were treated with AUTO-3 and 7 received the combination with pembrolizumab. There were no dose limiting toxicities (DLTs) and no treatment related deaths. Cytokine release syndrome (CRS) grade 1 occurred in 27% of patients and no higher-grade CRS was reported. There was only one case of grade 3 neurotoxicity. Early response rates are promising with an ORR of 57% (29% CR rate) in this ongoing trial [91].

Currently, there are several ongoing trials evaluating the combination of CAR-T cell treatment with infused ICI as shown in Table 2.

### 5.4. Built in CARs

The combination of CAR-T cell treatment with repetitive intravenous infusions of checkpoint blocking antibodies has several disadvantages, including (1) the need for repeated infusions with an associated increase in treatment cost, (2) the risk of immune related adverse events and (amongst other reasons) (3) lack of penetration of the antibody to the tumor site. Therefore researches have focused on building CARs that either secrete checkpoint inhibitors locally at the tumor site or build CARs with cell intrinsic checkpoint resistance.

### 5.5. CARs that Secrete Checkpoint Inhibitors In Situ

In 2016, Suarez et al. first reported on a built-in CAR in renal cell carcinoma (RCC) cell lines and in vivo xenograft models. They cloned an anti-PD-L1 antibody sequence into a bicistronic lentiviral vector encoding for an anti carbonic anhydrase IX (anti-CAIX) CAR-T cell. Treatment with this anti-PD-L1 secreting CAR-T cell led to a 50% decrease in T cell exhaustion markers (LAG-3, TIM-3 and PD-1) compared to treatment with a non-secreting CAR-T cell and a three times profounder reduction in tumor mass [92].

One year later, Li et al. reported similar outcomes with an anti-CD-19/PD-1 secreting CAR-T cell. After co-cultering these CAR-T cells with H292-CD19 or SKOV3-CD19 target cells (both with high PD-L1 expression), they found that after 24 h the IFNγ production was similar between the anti-CD19 CAR and the anti-CD19/PD-1 secreting CAR. However, after 72 h, IFNγ production was markedly higher in the anti-CD19/PD-1 secreting CAR indicating prolongation of effector functions. Furthermore, the proliferation rate upon antigen recognition was higher for the anti-CD-19/PD-1 secreting CAR and PD-1 expression was lower, indicating higher proliferative potential and protection from exhaustion. In a xenograft model using the same tumor cell lines, they were able to show, that the anti-CD19/PD-1 secreting CAR had better anti-tumor activity than either the anti-CD19 CAR alone or the anti-CD19 CAR combined with an infused PD-1 antibody. In addition, in vivo expansion was best for the anti-CD19/PD-1 secreting CAR-T cell compared to the other two modalities [93].

Rafiq et al. made similar observations in a mouse lymphoma and ovarian cancer cell model. They generated a second-generation CAR targeting either CD19 or MUC16 that secretes a PD-1 blocking scFV of an antibody. This led to an autocrine binding of the PD-1 antibody to the CAR-T cell but also to bystanding T cells. Similar to the studies above, the authors were able to show an increase in survival in mice treated with anti-PD-1 secreting CAR-T cell compared to CAR-T cell treatment alone [94].

### 5.6. Inhibiting Checkpoint Signaling in the CAR-T Cell

Another approach is to engineer the PD-1 receptor in order to inhibit intracellular signaling in the CAR-T cell. As an example, Chen et al. genetically engineered CAR-T cells to overexpress a PD-1 dominant negative receptor lacking the intracellular signaling domain. This CAR-T cell exhibited increased proliferation, cytotoxicity, better tumor control and prolonged survival in their mesothelioma mouse model compared to non-engineered CAR-T cells [95]. Using CRISPR/Cas9 technology, Rupp et al. generated PD-1 deficient anti-CD19 CAR-T cells. PD-L1 positive tumor cells rendered their normal CD19 CAR-T cells hypofunctional. With the use of PD-1 deficient CAR-T cells they were able to show enhanced tumor cell killing in a xenograft model of CD19 and PD-L1 positive AML. All animals that received the PD-1 deficient CAR-T cells cleared the tumors within 28 days, whereas this was only achieved in 17% of the mice treated with control CAR-T cells [96].

With the goal of overcoming PD-L1 effects on CAR-T cells, Hu et al. evaluated an anti-mesothelin second generation CAR-T cell with knocked down PD-1 against mesothelin positive triple negative breast cancer (TNBC) cells. They generated mesothelin targeting 4.1BB CAR-T cells that were able to kill mesothelin positive TNBC cells in vitro. As a next step, they compared PD-1 positive anti-mesothelin CAR-T cells to the same CAR-T cells after PD-1 disruption using CRISP/Cas9. The PD-1 disrupted CAR-T cells showed significantly higher antitumor activity in vitro (indicated by higher IFNγ and IL-2 production and cytotoxicity). Interestingly, they also added an anti-PD-1 antibody to rescue the PD-1 positive CAR-T cells. However, PD-1 disruption by CRISP/Cas9 exhibited higher cytotoxicity than the combination of an antibody plus PD-1 positive CAR-T cells. This effect was also observed in a mouse xenograft model, where treatment with PD-1 disrupted CARs lead to a significantly higher reduction of tumor burden than the other combination strategies [97].

Others used this technology to knockout different checkpoint molecules. As an example, Zhang et al. generated LAG-3 deficient CD19 CAR-T cells. These CAR-T cells displayed robust antigen-specific anti-tumor activity in cell culture and in murine xenograft models. However, the anti-tumor effects of the LAG-3 knockout CD19 CAR-T cells were similar to standard CAR-T cells, probably indicating that LAG-3 is not the primary checkpoint by which lymphoma cells induce T cell exhaustion [98]. Therefore, Zou et al. explored whether simultaneous knockout of three checkpoint molecules (PD-1, TIM-3 and LAG-3) in CAR-T cells targeting HER2 leads to increased efficacy. They were able to show that knock down of all three inhibitory receptors led to the highest cytotoxicity and IFNγ production compared to CAR-T cells with knockdown of one of the receptors or no knockdown. Furthermore, they were able to show that the triple knockdown CAR-T cells up-regulated CD56, which correlated with enhanced infiltration of the CAR-T cells into the tumor tissue [99].

Currently, most CAR-T cell treatment is done with autologous T cells, which is a time consuming and costly method. Therefore, Ren et al. used multiplex genome editing using CRISPR/Cas9 to generate allogeneic CAR-T cells with disrupted PD-1, TCR and human leukocyte antigen (HLA)-I against different target antigens. In a mouse xenograft prostate cancer model, they were able to show that PD-1 disrupted CAR-T cells displayed significantly enhanced antitumor activity compared to regular CAR-T cell therapy. They did not observe relevant alloreactivity or graft-versus-host disease (GVHD) with the allogeneic TCR and HLA-I deficient CAR-T cells, showing proof of concept of this approach [100].

### 5.7. Targeting the Microenvironment

Another possibility is to target the microenvironment with the CAR-T cell. As an example, Zhao et al. constructed a bispecific CAR-T cell that targets the human trophoblast cell surface antigen (Trop2) and PD-L1 at the same time in a gastric cancer model. They showed that the Trop2/PD-L1 CAR-T cells specifically killed Trop 2 and PD-L1 positive gastric cancer cells. The bispecific CAR produced much higher amounts of IFNγ than either the Trop2 or PD-L1 CAR-T cells. This also translated in vivo. The bispecific CAR inhibited tumor growth better than either of the single-target CAR-T cells [101]. Another very exciting approach undertaken by Xie et al. is to generate nanobody-based CAR-T cells that target the tumor microenvironment directly. The variable regions of heavy-chain-only antibodies (VHHs or nanobodies) are small, stable single-domain antibody fragments with affinities comparable to traditional scFVs that can access antigens differently due to their small size. They constructed such nanobody-based CAR-T cells against different molecules found in the tumor microenvironment. Using an anti-PD-L1 CAR they could reduce tumor growth in a melanoma xenograft model. In addition, they generated a CAR-T cell targeting the tumor stroma and vasculature through the EIIIB+ fibronectin splice variant, which is expressed by multiple tumor types. These CAR-T cells successfully delayed tumor growth and improved survival. These results form the basis for different combination strategies in the future [102].

Liu et al. generated chimeric switch receptor CAR-T cells that contain the extracellular domain of PD-1 fused to the transmembrane and cytoplasmic domain of CD28. When this switch receptor binds to PD-L1 it transmits an activating signal via CD28 instead of an inhibitory signal seen with the normal PD-1/PD-L1 interaction. When co-culturing these CAR-T cells with PD-L1 positive tumors, they observed an increased killing efficacy compared to CARs without the switch receptor, indicating that addition of a switch receptor can convert an inhibitory signal into an activating signal [103].

All these fascinating preclinical experiments are translated into in-human use with a number of early phase clinical trials that have been opened recently as summarized in Table 3.

CAR-T cell treatment has revolutionized the treatment of hematologic malignancies. As we are gaining more knowledge of mechanisms that are responsible for treatment failure and with the advances in genetic engineering strategies to overcome resistance are being explored. Results of clinical trials evaluating approaches as outlined in this review are eagerly awaited and are likely to further improve treatment outcomes especially in the field of solid tumors.

## 6. Toxicity Associated with Immune-Checkpoint Blockade Combination Strategies

The encouraging and exciting activity of ICI therapy comes at the cost of immune related adverse events (IRAE). IRAE are thought to arise from an “over-activation” of the immune system leading to autoimmune inflammatory events affecting virtually any organ, most commonly the skin, gastrointestinal tract, liver, endocrine system and lung [104,105]. Excellent guidelines on management of these conditions have been recently published and can be found elsewhere [106]. In this review, we have highlighted potential combination strategies with ICI. We have seen in the past that the combination of different ICI (i.e., nivolumab with ipilimumab) results in an increased rate of severe IRAE as reviewed recently in a meta-analysis [107]. Consequently, there is a relevant concern that the combination of ICI with abovementioned potent therapies may lead to excessive toxicity. Overall, clinical experience with the combination strategies discussed in this review is limited. However, a recent phase 3 trial comparing the combination of the tyrosine kinase inhibitor axitinib and pembrolizumab with sunitinib for the treatment of RCC reported an increased rate of grade 3-5 liver toxicity in the combination arm [15]. Similar evidence for the potential of additive toxicity comes from a phase II trial evaluating nivolumab in combination with ibrutinib for patients with advanced CLL [108]. In the latter study, diarrhea was the most commonly reported adverse event, probably indicating additive toxicity caused by the two drug classes. However, none of the eleven fatal adverse events that have been reported in this trial where deemed to be drug-related [108].

Regarding CAR-T cell treatment, the most relevant drug-specific adverse events are CRS and neurotoxicity [109]. Since CRS results from an over-activation of immune effector cells, combination with ICI causes significant concerns regarding excessive toxicity. Up to now, clinical experience with combination strategies of ICI and CAR-T cells is very limited. In the preliminary studies discussed above, the rate and severity of reported CRS was similar to CAR-T cell monotherapy and no life threatening CRS was reported. Overall, larger randomized studies will be required to evaluate the actual risk for severe adverse events with ICI combinations.

## 7. Predicting Response to Immune-Checkpoint Inhibition by Artificial Intelligence

### 7.1. Alterations in the Antigen Presenting Pathway

The cancer immunity cycle highlights a cascade of steps which are necessitated to produce anti-tumor responses by the immune system [110]. However, a magnitude of escape mechanisms prevent tumor neoantigen recognition and in turn abolish the effect of ICI. These escape mechanisms are found at the DNA level (e.g., loss of neoantigens due to chromosomal instability), at the RNA level (e.g., decreased neoantigen expression due to promoter hypermethylation) as well as at the protein level (e.g., gene mutations affecting HLA heterozygosity) [111]. Currently available and/or already established predictive markers for ICI such as PD-L1 [10,11,12,13] and tumor mutational burden (TMB) [112] only depict the tip of the iceberg of the cancer immunity cycle. Mutant tumor peptides have to be intracellularly processed into nine to eleven amino acid peptides, which must fit and be presented in the groove of one of the patients’ surface MHC I molecules [113]. Aspects of the MHC I processing and presentation pathway in order to predict tumor neoantigens, binding affinity of these tumor neoantigens to MHC I, as well as the TCR repertoire have come into the focus of immune-checkpoint blocking strategies. Despite a magnitude of evolving biomarkers for ICI and greatest interest in the gut microbiome [114], antibiotic treatment status [115,116] and T cell exhaustion markers [117], within this subsection we review the literature about tumor neoantigen presentation and prediction with regard to the application of ICI for cancer treatment.

A high TMB has been shown to be a positive predictive marker for clinical outcome with ICI across various tumor entities [118,119,120]. A higher tumor neoantigen burden is associated with improved clinical outcome in advanced NSCLC [120] and advanced melanoma [121] patients undergoing immune-checkpoint blockade and shows a strong correlation with TMB. However, mounting evidence suggests that especially patients with a high clonal neoantigen burden and a low intratumoral neoantigen heterogeneity benefit from ICI [122].

Among 77,803 identified tumor neoantigens, Rizvi et al. only found 28 (0.04%) in more than one melanoma patient [120]. Comparable findings (99% unique neoantigens) were reported among gastrointestinal tumors [123]. These data corroborate that tumor neoantigens appear to be private events. Neoantigen binding to MHC I is the most selective step leading to peptide presentation. Only 3–4% of predicted tumor neoantigens turn out to be MHC I binders and in turn form neoepitopes [124,125]. Bjerregaard et al. investigated natural T cell responses to predicted tumor neoepitopes. Among 1948 predicted neopeptide-MHC I combinations from 13 publications, the vast majority showed a strong binding affinity to MHC I. However, only 53 neoepitopes (3%) were able to elicit T cell responses [126].

Tumor neoantigen prediction models (as summarized in Table 4) could be of special interest for the application of ICI and key questions to be answered by these models are: which mutated proteins are processed into eight to eleven amino acid peptides by the proteasome, and are transferred into the endoplasmatic reticulum by the transporter associated with antigen processing (TAP), and are loaded onto one out of six MHC I molecules in the individual patient (about 12,000 HLA alleles identified in the human population [127]), and are shuttled to the cell surface by chaperone proteins in order to be recognized by cytotoxic T-lymphocytes [128].

Each of the aforementioned steps is crucial for proper tumor neoantigen presentation. Down-regulation of TAP1 (e.g., by promoter methylation) is associated with a lower infiltration of TILs and with an inferior clinical outcome in early colorectal cancer (CRC) [150] and genetic variants of TAP are associated with the development of high-grade cervical neoplasia [151]. Lower expression of HLA class I genes as well as of beta-2 microglobulin (β2m) are immune escape mechanisms in NSCLC [122,152,153] and melanoma [153] patients undergoing immune-checkpoint blockade. HLA class I loss has been shown to prevent continuous T cell recognition in a human melanoma model [154]. HLA-A down-regulation is mediated e.g., by the RNA-binding protein MEX3B [155], by loss of function mutations in the genes encoding the interferon-receptor associated Janus kinase 1 (JAK1) or Janus kinase 2 (JAK2) [156,157,158] or by truncating mutations in the gene encoding β2m [156]. A major impact of HLA class I genotype on clinical outcome with ICI has been corroborated by Chowell et al. HLA-I homozygosity in at least one locus was associated with an inferior survival in two independent cancer cohorts undergoing immune-checkpoint blockade and proved as an independent predictor of survival in multivariate analysis. The combined effect of HLA class I genotype and TMB on survival was greater than the effect of TMB alone [16]. In a similar approach, Goodman et al. reported a better discrimination of survival among TMB high cancer patients undergoing immune checkpoint blockade by considering the MHC I genotype [17]. Prediction models such as the Loss of Heterozygosity in Human Leukocyte Antigen (LOHHLA) bioinformatics tool enable estimation of allele-specific HLA loss from sequencing data and improve neoantigen prediction accuracy [146]. Hopkins et al. examined the role of the peripheral TCR repertoire in immunotherapy treated pancreatic adenocarcinoma. A low baseline clonality as well as a high number of expanded clones following treatment with anti-CTLA-4 targeting ipilimumab was associated with a statistically significantly longer survival. The latter results were not reproducible with anti-PD-1 targeting therapy [159]. Comparable findings concerning TCR repertoire dynamics [160] and clinical outcome [160,161] with anti-CTLA-4 and anti-PD-1 targeting therapy were reported in advanced melanoma patients [160]. Despite the limited number of patients included in the aforementioned retrospective analyses, the opposite impact of baseline TCR clonality on clinical outcome with anti-CTLA-4 and anti-PD-1 targeting therapy is hypothesis generating and suggests sequential immunotherapy strategies of anti-CTLA-4 followed by anti-PD-1 targeting therapy.

A high false positive rate remains a major drawback of tumor neoantigen prediction algorithms. MHC class I binding affinity (calculated as the wild-type peptide binding affinity relative to the mutant peptide binding affinity) was demonstrated to be a major determinant of cancer peptide immunogenicity and outperformed TMB as well as neoantigen burden for clinical outcome in melanoma and NSCLC patients undergoing immune-checkpoint blockade [162]. In an integrative approach, Kalaora et al. combined whole-exome and RNA sequencing with MHC-peptidomics (analysis of peptide binding to MHC I by liquid chromatography and tandem mass spectrometry) and the neoantigen prediction tool NETMHCpan in advanced melanoma patients. In a direct comparison, this prediction tool, which integrates binding affinity data and mass spectrometry data, outperformed other neoantigen prediction alogorithms [144]. The latter approach highlights the advantage of combining bioinformatic neoantigen prediction with MHC-peptidomics in order to reduce the rate of false positive neoepitopes, especially in cases of rare HLA allotypes [125,163].

However, peptides with a predicted high MHC I binding affinity are not necessarily immunogenic. In neoepitope prediction strategies, attempts such as the integration of information concerning the hydrophobicity of the TCR contact region [149,164], amino acid characteristics [140] or binding differences between wild-type and mutant epitopes [149] yield at increasing the probability to identify clinically relevant neoepitopes [149]. Calis et al. reported two common properties of neopeptide-MHC combinations, which cause differences in T cell recognition: (1) the composition of amino acids in the position 4-6 of the presented peptide as well as (2) the size and absence/presence of aromatic side chains [140]. Neopepsee, a machine-learning-based neoantigen prediction program, integrates nine immunogenicity features including the aforementioned features and was able to determine immunogenic neoantigens in melanoma and CLL. Furthermore, the presence of immunogenic neoantigens determined by Neopepsee was associated with a better prognosis in patients with gastric cancer [149]. Luksza et al. combined estimations of the probability that a neoantigen will be presented on MHC I and the probability that presented neoantigens will be recognized by the TCR repertoire based on tumor clonality, MHC I binding affinity and microbial epitope homology. This model was applied to two melanoma cohorts and one NSCLC cohort undergoing anti-CTLA-4 and anti-PD-1 targeting therapy, respectively, and predicted survival in each cohort [147]. Snyder et al. developed a bioinformatic pipeline incorporating MHC class I binding probability, TCR binding probability, patient specific HLA genotype and epitope-homology analysis in order to identify putative neoepitopes associated with clinical outcome in advanced melanoma patients undergoing anti-CTLA-4 targeting therapy. Among predicted neoantigens, conserved stretches of amino acids were identified that were shared by patients with clinical benefit exceeding six months. These neoepitope signatures were significantly associated with survival in the discovery as well as in the validation set [165]. Published studies evaluating the antigen presenting pathway and TCR repertoire by artificial intelligence and the impact on clinical outcome in patients undergoing immune-checkpoint blockade are summarized in Table 5.

A plethora of previous studies have focused on individual factors affecting the success of immune-checkpoint blockade in immuno-oncology. However, a comprehensive analysis incorporating multiple factors is of utmost importance. Apart from the antigen presenting pathway, future models predicting clinical outcome with ICI necessitate the integration of additional factors affecting the tumor-host interaction such as PD-L1 expression, gut microbiota composition, patient germline genetics, immune microenvironment composition as well as absence/presence of soluble inhibitory molecules as proposed in several cancer immunograms [117,166,167]. For such an approach, DNA sequencing data of the tumor, RNA sequencing data of the microenvironment and germline DNA sequencing will be required. In this regard, Xie et al. developed a multifactorial deep learning model integrating microsatellite instability (MSI-H) burden, somatic copy number alteration (SCNA) burden and modified TMB (mTMB) into four genomic clusters. Data were derived from 8,646 samples of The Cancer Genome Atlas (TCGA) across 29 tumor types. Interestingly, the abovementioned genomic features only showed a weak to moderate correlation, suggesting that each feature has a distinct impact on tumor biology. The authors used TCGA RNA sequencing data to characterize the tumor microenvironment of each genomic cluster by the level of TIL infiltration, expression of immune genes and status of immune pathways. Each cluster was associated with a unique immune landscape. Genomic clusters discriminated patients with different risk for OS in the entire cohort as well as in multiple cancer types. When applying these four genomic clusters to two anti-CTLA-4 treated melanoma cohorts, cluster 4 (MSIhigh, SCNAhigh, mTMBlow) showed the lowest rate of clinical benefit and the shortest OS [168].

However, prospective validation and reproducibility in a real-world setting will be prerequisites for applying such prediction models in clinical practice.

### 7.2. Radiomics

In general, the assessment of predictive biomarkers for ICI is frequently limited by the availability of tumor tissue, intralesional as well as interlesional tumor heterogeneity [169] and by expression dynamics during the course of disease [170] and necessitates invasive procedures with relevant periprocedural risks [171,172] in often comorbid cancer patients.

Due to the availability of routinely performed imaging studies and correlations of images with underlying biological processes radiomics may serve a new predictive tool in immuno-oncology in the near future. Apart from non-invasive identification of potential responders to ICI, addressing resistance mechanisms as well as visualization of drug distribution and of the tumor microenvironment are major goals of radiomics in immuno-oncology. Radiomics is based on common imaging modalities such as computed tomography (CT), positron emission tomography (PET) and magnetic resonance tomography (MRT) and necessitates the following steps: image acquisition, identification of the target volumes, segmentation, feature extraction and analysis [173].

#### 7.2.1. Assessment of Mutation Status by Radiomics

CT-based radiomic features are associated with molecular aberrations [174,175,176,177] in various types of cancer. Yang et al. found a highly statistically significant association between a CT-based radiomic signature and KRAS/NRAS/BRAF mutations in a test cohort of 61 CRC patients (area under curve (AUC): 0.869, *p* < 0.001) and confirmed the results in a validation cohort [174]. In the light of the recently reported positive predictive value of KRAS mutations for pembrolizumab monotherapy response in the KEYNOTE-042 study [178] such a radiomic approach could be of clinical relevance for treatment decisions in advanced non-squamous NSCLC. Mismatch repair deficient (dMMR) tumors harbor high numbers of mutation-associated neoantigens and are considered sensitive to ICI [179]. The latter finding in turn has led to the tissue/site-agnostic approval of pembrolizumab in dMMR solid tumors by the FDA. Huang et al. demonstrated the feasibility to assess the mismatch repair status by a CT-based radiomic signature in a test cohort of 140 CRC patients (AUC: 0.914, *p* < 0.001) and confirmed the good discrimination in a validation cohort including 114 CRC patients (AUC: 0.702, *p* = 0.012) [175]. Due to the low frequency of dMMR solid tumors in advanced stages [179], this radiomic approach will only identify a minority of potential responders to ICI. NSCLC harboring activating EGFR mutations are insensitive to ICI monotherapy [180,181]. Yip et al. showed the potential of quantitative CT imaging to predict the EGFR mutation status in operable NSCLC patients in the perioperative setting (AUC: 0.67) [176]. Comparable findings based on FDG-PET CT imaging were described by Gevaert et al. in stage 1–4 NSCLC patients (AUC: 0.89) [177].

#### 7.2.2. PD-1/PD-L1 Expression and Heterogeneity Assessed by Radiomics

CT based radiomic features are capable of separating patients with NSCLC [182,183,184,185,186] as well as head and neck squamous cell carcinoma (HNSCC) [183] with differing risk profiles for survival. Furthermore, CT based radiomic approaches allow prediction of dichotomous PD-L1 expression on tumor cells (tumor proportion score: TPS) [184,185] and density of CD3+ [184] or CD8+ [187] TILs in NSCLC. Successful anti-PD-1/anti-PD-L1 receptor-ligand-pair imaging by PET scans in mice with subcutaneously injected melanoma cells was demonstrated by Hettich et al. [188]. In a similar approach, Xing et al. [189] and Niemeijer et al. [190] investigated the correlation between PD-L1/PD-1 expression based on single photon emission computed tomography (SPECT), PD-L1/PD-1 PET and PD-L1/PD-1 expression assessed by immunohistochemistry (IHC) in NSCLC patients. Xing et al. used the anti-PD-L1 antibody NM-01, site-specifically labeled with technetium-99m, for SPECT imaging in 16 NSCLC patients (including squamous and non-squamous histology) in order to correlate tumor uptake with PD-L1 IHC. Patients with a PD-L1 expression ≤1% demonstrated statistically significantly lower tumor to peripheral blood tracer uptake ratios (mean 1.89 vs. 2.49, *p* = 0.0048) with a corresponding AUC of 0.88. It is noteworthy that four out of twelve patients with lymph nodes metastases showed considerable intra-patient differences (>20%) of PD-L1 expression [189]. Niemeijer et al. reported a statistically significant correlation between radiotracer uptake (18F-BMS-986192, standardized uptake value: SUV) and PD-L1 expression based on IHC (PD-L1 ≥50%: SUVpeak 8.2 versus PD-L1 <50%: SUVpeak 2.9, *p* = 0.018). The observed heterogeneous intrapatient and interpatient radiotracer uptake highlights the challenge to adequately assess tumor PD-L1 expression by core needle biopsies [190]. The latter two studies prove the feasibility to assess locoregional differences of PD-L1 expression in primary tumors and distant metastases. The assessment of intrapatient PD-L1 expression heterogeneity by radiomics may facilitate treatment decisions concerning intensity of therapy (ICI monotherapy versus ICI combined with chemotherapy) in clinical practice.

#### 7.2.3. Radiomics Predict Clinical Outcome with ICI Therapy

By combining CT images and RNA-sequencing genomic data from tumor biopsies of patients with advanced solid tumors (MOSCATO trial) [191], Sun et al. developed a radiomic signature that could discriminate between high (>median) and low (<median) density of CD8+ TILs (AUC: 0.74, *p* < 0.0001) [187] and validated the findings in three independent advanced solid tumor cohorts: TCGA validation set [192], tumor immune phenotype validation set [193] and immunotherapy-treated validation set [194]. Patients with a high radiomic score (CD8+ TILs > median) showed a statistically significantly increased median OS (24.3 versus 11.5 months, *p* = 0.0081) in the immunotherapy-treated validation set and the radiomic score proved to be the strongest independent prognosticator for OS in multivariate analysis (hazard ratio (HR): 0.52, *p* = 0.0022) [187]. Bensch et al. found a better correlation between clinical outcome and PD-L1 status assessment by PET imaging (89Zr-atezolizumab) in comparison to PD-L1 evaluation by IHC or RNA-sequencing data in 22 patients undergoing treatment with atezolizumab for bladder cancer, NSCLC or TNBC [195]. Khorrami et al. evaluated changes in the radiomic texture during two to three cycles of ICI therapy and reported the “delta-radiomic risk-score“ to predict response as well as OS with ICI in NSCLC [196]. Trebeschi et al. developed a radiomic signature based on pre-treatment CT images on a lesional level in advanced NSCLC and melanoma patients undergoing anti-PD-1 therapy. These radiomic features were significantly associated with response in pulmonary and nodal NSCLC metastases, whereas the model performed poorly on pulmonary and hepatic melanoma metastases. However, the model statistically significantly predicted OS in both tumor types (NSCLC: AUC: 0.76, *p* < 0.01; melanoma: AUC: 0.77, *p* < 0.01) [197]. Correlations of CT-based radiomic features and therapy response were also reported for patients with advanced ovarian cancer [198] and bladder cancer [199] undergoing immune-checkpoint blockade. Table 6 summarizes radiomics studies predicting clinical outcome with immune-checkpoint blockade.

On the one hand, a subset of advanced cancer patients derives long-term survival from immune-checkpoint blockade, on the other hand, up to nine per cent of patients experience hyperprogressive disease with rapid fatal outcome upon initiation of anti-PD-1/anti-PD-L1 therapy [203]. In a clinical-radiomic approach Tunali et al. were able to identify patients with a time to progression < 2 months or hyperprogressive disease within an advanced NSCLC cohort treated with single agent or double agent immunotherapy [200]. The latter finding is of utmost importance in clinical practice as such cancer patients should not be treated with ICI monotherapy or with ICI at all. Apart from predicting clinical outcome with immunotherapy, radiomics also has the potential to predict immune-related adverse event. In a small series of 32 advanced cancer patients, Colen et al. found radiomic features that identified the two patients who experienced immunotherapy-induced pneumonitis (accuracy: 100%, *p* = 0.0033) [204].

The abovementioned findings corroborate the potential of radiomics to visualize drug distribution, tumor characteristics as well as tumor heterogeneity and the feasibility to predict clinical outcome with ICI. However, a major caveat remains the standardization of imaging acquisition, validation in prospective clinical trials and reproducibility in a real-world setting. ICI trials in advanced solid tumors such as the “INSPIRE” trial (NCT02644369) are prospectively investigating changes in radiomic imaging parameters as well as correlations between tumor genomic profiles and radiomic imaging signatures.

## 8. Conclusions

ICI represent a promising therapeutic strategy to overcome T cell exhaustion in order to reinvigorate T cell responses against cancer cells. CTLA-4, PD-1 and PD-L1 are the most extensively investigated and targeted immune-checkpoints, however, several other immune-checkpoint molecules such as LAG-3 and TIM-3 are therapeutically targeted in ongoing trials. In recent years, a magnitude of ICI has been approved as monotherapy or as combination therapy for the treatment of solid and hematologic malignancies. Combination strategies as for example with tyrosine kinase inhibitors in metastatic RCC improve clinical outcome but come at the cost of increased grade 3–5 hepatotoxicity. CDK4/6 inhibitors, which are considered as therapeutic standard in combination with endocrine therapy in metastatic hormone receptor positive breast cancer, have been demonstrated to increase antigen presentation in cancer cells and as consequence might serve as potent combination drugs for ICI.

The success with CAR-T cells in hematologic malignancies has revolutionized the therapeutic landscape in DLBCL and B-ALL. Up-regulation of immune-checkpoints drives resistance to CART-T cell therapy in hematologic and solid malignancies, which can be overcome by combination strategies with ICI without increasing CRS or neurotoxicity rates. The latter therapeutic approach can be accomplished by separately infusing ICI, by CAR-T cells that secrete checkpoint inhibitors locally as well as by CAR-T cells with cell intrinsic checkpoint resistance.

Although there is a positive correlation between TMB and survival with ICI across various tumor types, high TMB does not necessarily result in immunogenicity. A plethora of steps is crucial for proper tumor neoantigen presentation and T cell recognition. Alterations in the antigen presenting pathway give rise to resistance mechanisms that in turn abolish the effect of ICI. Tumor neoantigen prediction models have been shown to identify cancer patients who benefit most from immune-checkpoint blockade. However, a high false positive rate is a drawback of these models. Individual immunograms including tumor neoantigen prediction, factors affecting the tumor-host interaction such as PD-L1 expression, gut microbiota composition, patient germline genetics, immune microenvironment composition as well as absence/presence of soluble inhibitory molecules may help to distinguish responders from non-responders to ICI.

The predictive value of already established biomarkers such as PD-L1 is considerably heterogeneous across various malignancies and negativity does not exclude responses. Furthermore, intratumoral and intrapatient heterogeneity complicate tumor tissue-based biomarker assessment. Radiomics offers the opportunity to evaluate biomarkers (including intrapatient heterogeneity) based on imaging studies without the necessity to perform tumor tissue biopsies. Several radiomics studies have shown to predict clinical outcome with ICI. Radiomics might also help to identify patients who are at risk for hyperprogressive disease upon initiation of anti-PD-1/anti-PD-L1 therapy and patients who are at risk for high grade IRAE. However, standardization of imaging acquisition and validation of findings in prospective clinical trials will be necessitated before implementation in clinical practice.

## Figures and Tables

**Table 1:** Expression pattern of PD-1 and its ligands. Adapted from [43,45].

Protein (Gene)	Binding Protein	Expression Pattern
PD-1 (*PDCD1*)	PD-L1 and PD-L2	Activated T cells, maturing thymocytes, B cells, NK cells, NKT cells, myeloid and APC subsets and innate lymphoid cell progenitors Some cancer cells
PD-L1 (*CD274*)	PD-1 and CD80	APCs, T cells and B cells Thymic cortex some non-hematopoietic lineages some cancer cell lineages
PD-L2 (*PDCD1L2*)	PD-1 and RGMb	APCs, some B cells, some mast cells and TH2 cells Thymic medulla Some cancer cells

PD-1: programmed cell death protein 1, PD-L1/2: programmed cell death-ligand 1/2, CD: cluster of differentiation, RGMb: repulsive guidance molecule B, APC: antigen presenting cell, TH2: T helper 2 cell, NKT cell: natural killer T cell, NK cell: natural killer cell.

**Table 2:** Ongoing trials with chimeric antigen receptor (CAR)-T cells in combination with infused checkpoint inhibitors (www.clinicaltrials.gov, access date: 01/30/2020).

NCT Number	Disease	Treatment	Country
NCT04003649	Glioblastoma	IL13R alpha 2 CAR-T cells +/- nivolumab and ipilimumab	USA
NCT03726515	Glioblastoma	EGFRvIII CAR-T cells + pembrolizumab	USA
NCT04205409	CLL, DLBCL, follicular lymphoma	nivolumab after CD19 CAR-T cells	USA
NCT03310619	B cell malignancies	CD19 CAR-T cells (JCAR017) + durvalumab	USA
NCT02706405	B-NHL	CD19 CAR-T cells (JCAR014) + durvalumab	USA
NCT02926833	DLBCL	Axi-cel + atezolizumab	USA
NCT03630159	DLBCL	Tisa-cel + pembrolizumab	USA
NCT02650999	DLBCL, MCL follicular lymphoma	pembrolizumab after CD19 CAR-T cell failure	USA
NCT04134325	Hodgkin’s lymphoma	PD-1 Inhibitors after CD30 CAR-T cell failure	USA

NCT number: ClinicalTrials.gov identifier, (CAR)-T cell: chimeric antigen receptor T cell; CLL: chronic lymphocytic leukemia; DLBCL: diffuse large B cell lymphoma; B-NHL: B cell Non-Hodgkin lymphoma; MCL: mantle cell lymphoma.

**Table 3:** Overview of clinical trials with genetically engineered CAR-T cells (www.clinicaltrials.gov, access date: 01/30/2020).

NCT Number	Disease	Treatment	Country
NCT04213469	B cell lymphoma	CD19 CAR-T cells with PD-1 knockout	China
NCT04162119	Multiple myeloma	BCMA-PD-1 secreting-CAR-T cells	China
NCT03932955	B cell lymphoma	CD19/PD-1 bispecific CAR-T cells	China
NCT03706326	Esophageal cancer	MUC1 CAR-T cells with PD-1 knockout	China
NCT03672305	Hepatocellular carcinoma	c-Met/PD-L1 bispecific CAR-T cells	China
NCT03615313	Advanced solid tumors, mesothelin positive	Mesothelin-PD-1 secreting CAR-T cells	China
NCT03540303	B cell lymphoma	CD19/PD-1 secreting-CAR-T cells	China
NCT03525782	NSCLC	MUC1 CAR-T cells with PD-1 knockout	China
NCT03208556	B cell lymphoma	CD19 CAR-T cells with cell intrinsic shRNA based PD-1 inhibition	China
NCT03182816	Advanced solid tumors	EGFR CAR-T cells with anti-CTLA-4/PD-1 secretion	China
NCT03182803	Advanced solid tumors	Mesothelin CAR-T cells with anti-CTLA-4/PD-1 secretion	China
NCT03179007	Advanced solid tumors	MUC1 CAR-T cells with anti-CTLA-4/PD-1 secretion	China
NCT03030001	Advanced solid tumors	Mesothelin-PD-1 secreting CAR-T cells	China
NCT02937844	Glioblastoma	Anti-PD-L1 chimeric switch receptor CAR-T cells	China
NCT02862028	Advanced solid tumors, EGFR positive	EGFR-PD-1 secreting CAR-T cells	China

NCT number: ClinicalTrials.gov identifier, NSCLC: non-small cell lung cancer, EGFR: epidermal growth factor receptor, CAR-T cell: chimeric antigen receptor T cell, PD-1: programmed cell death protein 1, BCMA: B cell maturation antigen, CD: cluster of differentiation, MUC1: Mucin 1, shRNA: small hairpin RNA, CTLA-4: cytotoxic T-lymphocyte protein 4.

**Table 4:** Overview of (tumor) neoantigen prediction models.

Reference	Publication Date	Author
[129]	1998	Mamitsuka et al.
[130]	2002	Dönnes et al.
[131]	2003	Nielsen et al.
[132]	2005	Larsen et al.
[133]	2006	Antes et al.
[134]	2007	Nielsen et al.
[135]	2008	Lundegaard et al.
[136]	2009	Hoof et al.
[137]	2009	Zhang et al.
[138]	2009	Kim et al.
[139]	2011	Lundegaard et al.
[140]	2013	Calis et al.
[125]	2014	Yadav et al.
[141]	2015	Pedersen et al.
[142]	2016	Andreatta et al.
[143]	2016	Nielsen et al.
[144]	2016	Kalaora et al.
[145]	2017	Jurtz et al.
[146]	2017	McGranahan et al.
[147]	2017	Luksza et al.
[148]	2018	O’Donnell et al.
[149]	2018	Kim et al.

**Table 5:** Impact of the antigen presenting pathway and T cell receptor (TCR) repertoire on clinical outcome with immune-checkpoint inhibitors (ICI).

Reference	Author	Tumor Entity	Findings
[122]	McGranahan et al.	NSCLC, melanoma	↑ PFS/OS with high clonal neoantigen burden + low intratumoral neoantigen heterogeneity
[152]	Gettinger et al.	NSCLC	β2m loss drives resistance to ICI
[153]	Sade-Feldman et al.	melanoma	β2m LOH drives resistance to ICI
[16]	Chowell et al.	solid tumors	↑ OS with maximal heterozygosity at HLA-I loci
[17]	Goodman et al.	solid tumors	↑ ORR/PFS/OS prediction by MHC I genotype analysis among TMBhigh tumors
[159]	Hopkins et al.	pancreatic ductal adenocarcinoma	↑ OS with low baseline TCR clonality before anti-CTLA-4 Tx ↑ OS with higher number of expanded TCR clones following anti-CTLA-4 Tx
[161]	Hogan et al.	melanoma	↑ ORR/PFS with low baseline TCR clonality in anti-CTLA-4 treated patients ↑ ORR/PFS with high baseline TCR clonality in anti-PD-1 treated patients
[162]	Ghorani et al.	NSCLC, melanoma	↑ PFS/OS prediction by assessment of differential binding affinity of mutated peptides for MHC I compared to TMB or tumor neoantigen burden
[147]	Luksza et al.	NSCLC, melanoma	OS discrimination based on neoantigen MHC I binding affinity and T cell recognition
[165]	Snyder et al.	melanoma	OS prediction based on neoantigen MHC I binding probability, TCR binding probability, HLA genotype and epitope-homology analysis

PFS: progression-free survival; OS: overall survival; MHC: major histocompatibility complex; TCR: T cell receptor; HLA: human leukocyte antigen; ORR: overall response rate; NSCLC: non-small cell lung cancer; β2m: beta-2 microglobulin; ICI: immune-checkpoint inhibitor; LOH: loss of heterzygosity; TMB: tumor mutational burden; CTLA-4: cytotoxic T-lymphocyte protein 4; Tx: therapy; PD-1: programmed cell death protein 1;.

**Table 6:** Prediction of clinical outcome by radiomics in cancer patients undergoing immune-checkpoint blockade.

Reference	Author	Tumor Entity	Findings
[187]	Sun et al.	solid tumors	OS prediction based on radiomics CD8+ cell score
[195]	Bensch et al.	bladder cancer, NSCLC, TNBC	↑ ORR/PFS/OS prediction by PET evaluation with zirconium-89-labeled atezolizumab compared to IHC or RNA-sequencing based PD-L1 assessment
[196]	Khorrami et al.	NSCLC	ORR and OS prediction based on changes in radiomic texture (“DelRADx”)
[197]	Trebeschi et al.	melanoma, NSCLC	Response prediction of individual metastases and OS prediction based on multiple radiomic features
[198]	Himoto et al.	ovarian cancer	Prediction of clinical benefit by intratumoral heterogeneity (radiomic feature) and by number of disease sites
[199]	Ligero et al.	solid tumors	↑ ORR prediction by clinical-radiomics signature score
[200]	Tunali et al.	NSCLC	Prediction of hyperprogressive disease based on clinical-radiomic models
[201]	Dercle et al.	non-squamous NSCLC	PFS prediction based on tumor volume reduction, infiltration of tumor boundaries or spatial heterogeneity
[202]	Korpics et al.	solid tumors	Prediction of local tumor failure, PFS and OS in cancer patients receiving SBRT and anti-PD-1 Tx based on a radiomics score

PET: positron emission tomography; PFS: progression-free survival; SBRT: stereotactic body radiotherapy, Tx: therapy; NSCLC: non-small cell lung cancer; TNBC: triple negative breast cancer; OS: overall survival; ORR: overall response rate; IHC: immunohistochemistry; PD-L1: programmed cell death-ligand 1.

## Abbreviations

AB	antibody
Akt	protein kinase B
AML	acute myeloid leukemia
APC	antigen presenting cell
AUC	area under curve
β2m	beta-2 microglobulin
B-ALL	B cell acute lymphoblastic leukemia
BCMA	B cell maturation antigen
BCR	B cell receptor
BTK	Bruton’s tyrosine kinase
CAIX	carbonic anhydrase IX
CAR	chimeric antigen receptor
CD	cluster of differentiation
CDK4/6	cyclin dependent kinase 4 and 6
CLL	chronic lymphocytic leukemia
CR	complete remission
CRC	colorectal cancer
CRS	cytokine release syndrome
CT	computed tomography
CTLA-4	cytotoxic T-lymphocyte protein 4
CTx	chemotherapy
DLBCL	diffuse large B cell lymphoma
DLT	dose limiting toxicity
dMMR	mismatch repair deficiency
EGFR	epidermal growth factor receptor
EGFRvIII	epidermal growth factor receptor variant III
ERK	extracellular signal-regulated kinase
FDA	Food and Drug Administration
GD2	disialoganglioside
GVHD	graft-versus-host disease
GSK-3	serine/threonine kinase glycogen synthase kinase 3
HCC	hepatocellular carcinoma
HER2	human epidermal growth factor receptor 2
HL	Hodgkin’s lymphoma
HLA	human leukocyte antigen
HNSCC	head and neck squamous cell carcinoma
HR	hazard ratio
ICI	immune-checkpoint inhibitors
IHC	immunohistochemistry
IL	interleukin
IFNγ	interferon-gamma
IRAE	immune-related adverse events
ITK	interleukin-2 inducible T cell kinase
ITIM	immunoreceptor tyrosine-based inhibitory motif
ITSM	immunoreceptor tyrosine-based switch motif
JAK	Janus kinase
LAG-3	lymphocyte-activation gene 3
LAT	linker for activation of T cells
Lck	lymphocyte-specific protein tyrosine kinase
LOH	loss of heterozygosity
LOHHLA	Loss of Heterozygosity in Human Leukocyte Antigen
MAPK	mitogen-activated protein kinase
MCL	mantle cell lymphoma
MHC	major histocompatibility complex
MRT	magnetic resonance tomography
MSI-H	microsatellite instability
mTOR	mechanistic target of rapamycin
MUC1	mucin 1
NFAT	nuclear factor of activated T cells
NK cell	natural killer cell
NKT cell	natural killer T cell
NFKB	nuclear factor kappa-light-chain-enhancer of activated B cells
NHL	non-Hodgkin lymphoma
NR2F6	nuclear receptor subfamily 2 group F member 6
NSCLC	non-small cell lung cancer
OS	overall survival
ORR	overall response rate
PD-1	programmed cell death protein 1
PD-L1	programmed cell death-ligand 1
PET	positron emission tomography
PFS	progression-free survival
PI3K	phosphatidylinositol 3-kinase
PKC	protein kinase C
PLCg	phospholipase C gamma 1
PMBCL	primary mediastinal B cell lymphoma
PR	partial remission
RCC	renal cell carcinoma
RGMb	repulsive guidance molecule B
SBRT	stereotactic body radiotherapy
scFV	single-chain variable fragment
SCNA	somatic copy number alterations
SHP	small heterodimer partner
shRNA	small hairpin RNA
SPECT	single photon emission computed tomography
SUV	standardized uptake value
TAP	transporter associated with antigen processing
tbx21	T-box transcription factor 21
TCGA	The Cancer Genome Atlas
TCR	T cell receptor
TFH	T follicular helper cell
TFR	T follicular regulatory cell
TH2	T helper 2 cell
TILs	tumor infiltrating lymphocytes
TIM-3	T cell membrane protein 3
TKI	tyrosine kinase inhibitor
TMB	tumor mutational burden
TNBC	triple negative breast cancer
TNFα	tumor necrosis factor alpha
TOX	thymocyte selection-associated high mobility group box protein TOX
TPS	tumor proportion score
Treg	regulatory T cell
Tx	therapy
VEGF	vascular endothelial growth factor
VEGFR	vascular endothelial growth factor receptor
ZAP70	zeta chain-associated protein kinase 70
